# Tertiary lymphoid structures in renal cell carcinoma: from heterogeneity dissection to translational precision immunotherapy

**DOI:** 10.3389/fimmu.2025.1732056

**Published:** 2026-01-29

**Authors:** Zhexian Li, Sensen Ruan, Yang Yu, Hao Xiang, Wenjie Mi

**Affiliations:** 1 Department of Urology, The Second Affiliated Hospital of Dalian Medical University, Dalian, Liaoning, China; 2 Dalian Medical University, Dalian, Liaoning, China

**Keywords:** heterogeneity, immunotherapy, renal cell carcinoma, tertiary lymphoid structure (TLS), tumor microenvironment

## Abstract

Renal cell carcinoma (RCC), characterized by its distinctive hypoxic and immunosuppressive tumor microenvironment (TME), demonstrates suboptimal responses to current immunotherapeutic interventions. Tertiary lymphoid structures (TLS), defined as ectopically organized immune cell aggregates that develop in non-lymphoid tissues, function as “plastic immune organs” and exhibit considerable promise as both prognostic indicators and therapeutic targets. Notably, TLS in RCC manifest significant heterogeneity, with specific subsets associated with favorable clinical outcomes while others correlate with adverse prognosis. This review systematically examines the cellular composition, formation, classification criteria, and evaluation methods of TLS in RCC, with particular emphasis on the relationship between TLS heterogeneity and differential prognostic implications. We further explore potential regulatory mechanisms underlying these divergent clinical outcomes and provide a comprehensive synthesis of current TLS-targeted therapeutic strategies, including recent clinical advancements. Finally, we delineate the prevailing challenges in TLS research and propose future directions. This work provides a theoretical foundation and research framework for future patient outcome-oriented prospective studies and clinical trials, while also offering insights for the development of TLS-related precision immunotherapy strategies.

## 1 Introduction

Renal cell carcinoma (RCC) represents the most prevalent histopathological subtype of renal malignancies. According to 2022 global cancer statistics, RCC ranks as the 14th most common malignancy worldwide, with annual new cases exceeding 400,000 (1, 2). Notably, approximately 30% of patients present with metastatic disease at initial diagnosis, while up to 50% of surgically treated localized RCC cases eventually develop distant metastases (3). In recent years, immunotherapy has emerged as an increasingly pivotal component in the comprehensive management of advanced RCC, with its clinical efficacy being closely associated with modulation of the tumor immune microenvironment (TIME).

In the tumor microenvironment (TME), all immune components are collectively defined as the TIME, which has been demonstrated to critically influence tumorigenesis, recurrence, and metastasis (4). The TIME exhibits profound heterogeneity in its cellular composition (5). The immunological landscape of RCC is characterized by three cardinal features: immune cell infiltration patterns, the expression of immunosuppressive factors, and regulatory networks of cytokines and chemokines, all of which collectively modulate tumor invasiveness, metastatic potential, and response to immunotherapy. Despite the transformative impact of immune checkpoint inhibitors (ICIs) on the treatment of advanced RCC, significant challenges persist in clinical practice. For instance, among patients with metastatic clear cell renal cell carcinoma (mccRCC), the objective response rate (ORR) to ICI monotherapy ranges from approximately 16% to 36%. While combination ICI therapies can increase ORRs to 40–60%, a substantial proportion of patients still fail to respond. Furthermore, some combination regimens have not demonstrated superiority over conventional treatments or are ineffective in patients resistant to immunotherapy. This highlights the limited response rates to ICI therapy and the presence of primary resistance (6). Currently, reliable tools for accurately predicting patients’ long-term survival outcomes or sustained therapeutic benefit are lacking in clinical settings. This reality underscores the urgent need for more biologically grounded auxiliary assessment indicators that can reflect the tumor’s immune status.

Furthermore, in current clinical practice, risk stratification and treatment response assessment for RCC still primarily rely on traditional indicators such as tumor node metastasis (TNM) staging, histopathological grading, and imaging examinations. These indicators mainly reflect tumor burden and anatomical features, making it difficult to capture the dynamic changes within the tumor immune microenvironment. In recent years, although the expression level of programmed death-ligand 1 (PD-L1), tumor mutational burden (TMB), and specific transcriptomic immune signatures have demonstrated certain predictive value in various solid tumors such as melanoma and non-small cell lung cancer (7–9), within the field of RCC, the stability, cross-cohort reproducibility, and consistency with clinical outcomes of these indicators remain limited. A biomarker system with broad consensus has yet to be established (10, 11). Consequently, current clinical decision-making still lacks sufficient capability to achieve personalized patient stratification and precise treatment selection based on the refined characteristics of the TIME.

Tertiary lymphoid structures (TLS), as aggregates of immune cells formed in non-lymphoid tissues, are believed to play a significant role in regulating local antitumor immune responses. Emerging evidence indicates that TLS within the TIME correlate with ICI responsiveness and may serve as both prognostic and predictive biomarkers for immunotherapy outcomes (12–14). Functioning as “plastic immune organs” (15, 16), TLS locally activate B-cell antibody secretion and T-cell clonal expansion across multiple malignancies, including RCC, gastric cancer, and pancreatic carcinoma (17–19), thereby exerting anti-tumor effects. Paradoxically, other RCC studies report TLS-associated enrichment of programmed death-ligand 1 (PD-L1) tumor-associated macrophages and regulatory T cells (Tregs) (20), fostering an immunosuppressive milieu that promotes tumor progression. Furthermore, while CD8^+^ T-cell infiltration typically correlates with improved prognosis in most cancers, RCC demonstrates the counterintuitive association of abundant CD8^+^ T cells with poorer outcomes (21). These findings reveal the dual nature of TLS in RCC: they may either potentiate anti-tumor immunity or paradoxically drive immunosuppression and tumor advancement. This dichotomy likely stems from intrinsic TLS heterogeneity encompassing cellular composition, maturation status, spatial distribution, and density. Such functional ambivalence may explain the current challenges in utilizing TLS detection to guide therapeutic decision-making for patients with advanced RCC.

Based on the above background, this review comprehensively examines the composition of TLS in RCC, the regulatory mechanisms underlying their formation, and current classification and evaluation methodologies. We highlight the critical impact of TLS heterogeneity on patient prognosis and elucidate the dual mechanisms through which TLS exert both anti-tumor effects and immunosuppressive functions in RCC. The therapeutic potential of TLS is explored from dual perspectives—both as prognostic biomarkers and therapeutic targets—with a systematic synthesis of existing TLS-targeted treatment approaches. Finally, we address current challenges in TLS research specific to RCC and extend this discussion to TLS in other malignancies, proposing key future research directions. This lays the foundation for future patient-outcome-oriented prospective studies and biomarker validation, with the anticipation that TLS can be utilized in clinical practice to develop more precise personalized immunotherapy strategies.

## 2 Composition and regulatory mechanisms of TLS neogenesis in RCC

### 2.1 Composition of TLS in RCC

**Figure 1:**
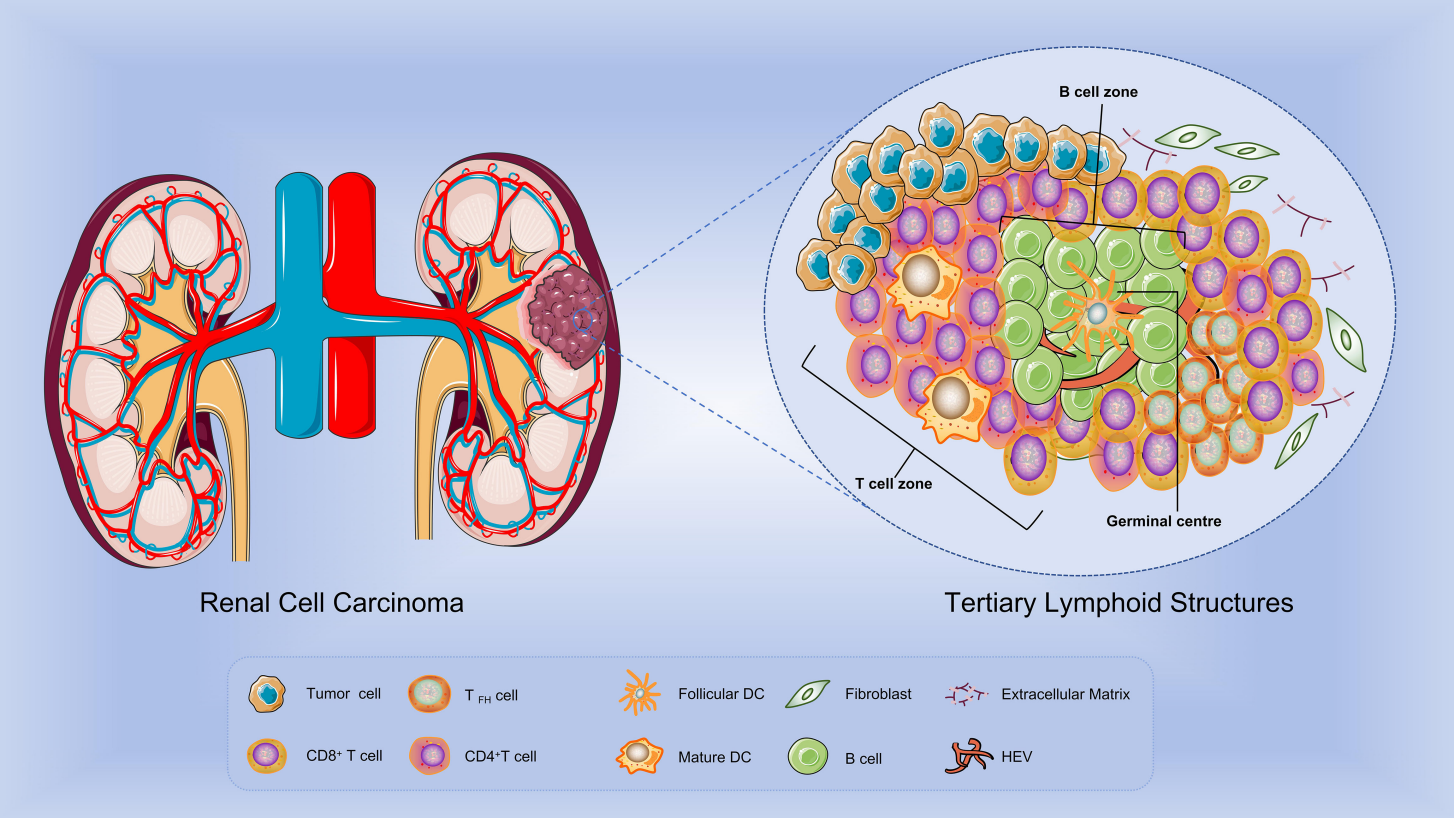
Composition of TLS in renal cell carcinoma. Fully mature tertiary lymphoid structures (TLS) are primarily composed of T-cell zones, B-cell zones, and high endothelial venules (HEV), exhibiting active germinal center (GC) reactions. Within the T-cell zone, CD4+ T cells, CD8+ T cells, and mature dendritic cells (DC) are predominantly present. The B-cell zone primarily consists of B cells and follicular dendritic cells (FDCs). HEV facilitate lymphocyte recruitment and trafficking to inflammatory sites.

TLS are organized aggregates of immune cells that develop postnatally in non-lymphoid tissues under pathological conditions, including autoimmune diseases, chronic infections, and cancer (22). Similar to most solid tumors, RCC-associated TLS exhibit progressive maturation, with its composition and structure becoming increasingly refined. Early TLS (E-TLS) primarily consist of dense lymphocyte clusters and abundant fibroblasts, lacking follicular dendritic cells (FDCs). At this stage, T cells and B cells are not compartmentalized, and CD21/CD23 signaling is absent. Primary follicle-like TLS (PFL-TLS), representing the initial stage of TLS maturation, contain immature FDCs but no observable germinal centers (GCs). T cells and B cells gradually segregate into distinct zones, exhibiting CD21^+^ but CD23^-^ immunophenotypes. Secondary follicle-like TLS (SFL-TLS), also referred to as mature TLS (**Figure 1**), are characterized by well-developed FDC networks, active GC reactions resembling secondary lymphoid organs (SLO), and clear spatial segregation of T-cell and B-cell zones (CD21^+^CD23^+^) (23, 24). Mature GCs consist of an outer light zone (LZ) and an inner dark zone (DZ), which serve as critical sites for B-cell maturation. Within these compartments, B cells undergo somatic hypermutation (SHM) and affinity-based selection, ultimately producing high-affinity antibodies (25). Mature TLS in RCC are composed of distinct structural regions, including a T-cell zone, a B-cell zone, and high endothelial venules (HEVs). The T-cell areas are predominantly populated by CD4^+^ T cells, CD8^+^ T cells, and mature dendritic cells (DCs). The B-cell areas primarily contain B cells and FDCs, with FDCs localized at the core of B-cell clusters, playing a crucial role in antigen presentation and B-cell activation. HEVs, which facilitate lymphocyte recruitment to inflammatory sites, are marked by peripheral node addressin (PNAd) and vascular addressin MECA79 (23). Additionally, fibroblastic reticular cells (FRCs) are frequently observed at the periphery of TLS, contributing to stromal support and the retention of immune cells.

### 2.2 Regulatory mechanisms of TLS formation in RCC

The precise mechanisms driving TLS formation in cancer remain incompletely understood. However, given that TLS structurally and functionally resemble SLO, insights into TLS formation in RCC may be gleaned from studying SLO development. SLO, including lymph nodes, spleen, Peyer’s patches, and mucosa-associated lymphoid tissue (MALT), form during embryogenesis or the initial postnatal weeks through highly coordinated interactions among hematopoietic cells, non-lymphoid stromal cells, adhesion molecules, chemokines, cytokines, as well as growth and survival factors (26, 27). TLS formation follows similar principles, with a key distinction being that TLS in most tissues lack a capsule, resulting in direct exposure to the tumor cells. Notably, in most tumor tissues, these unencapsulated TLS can persist stably and even influence patient prognosis.

**Figure 2:**
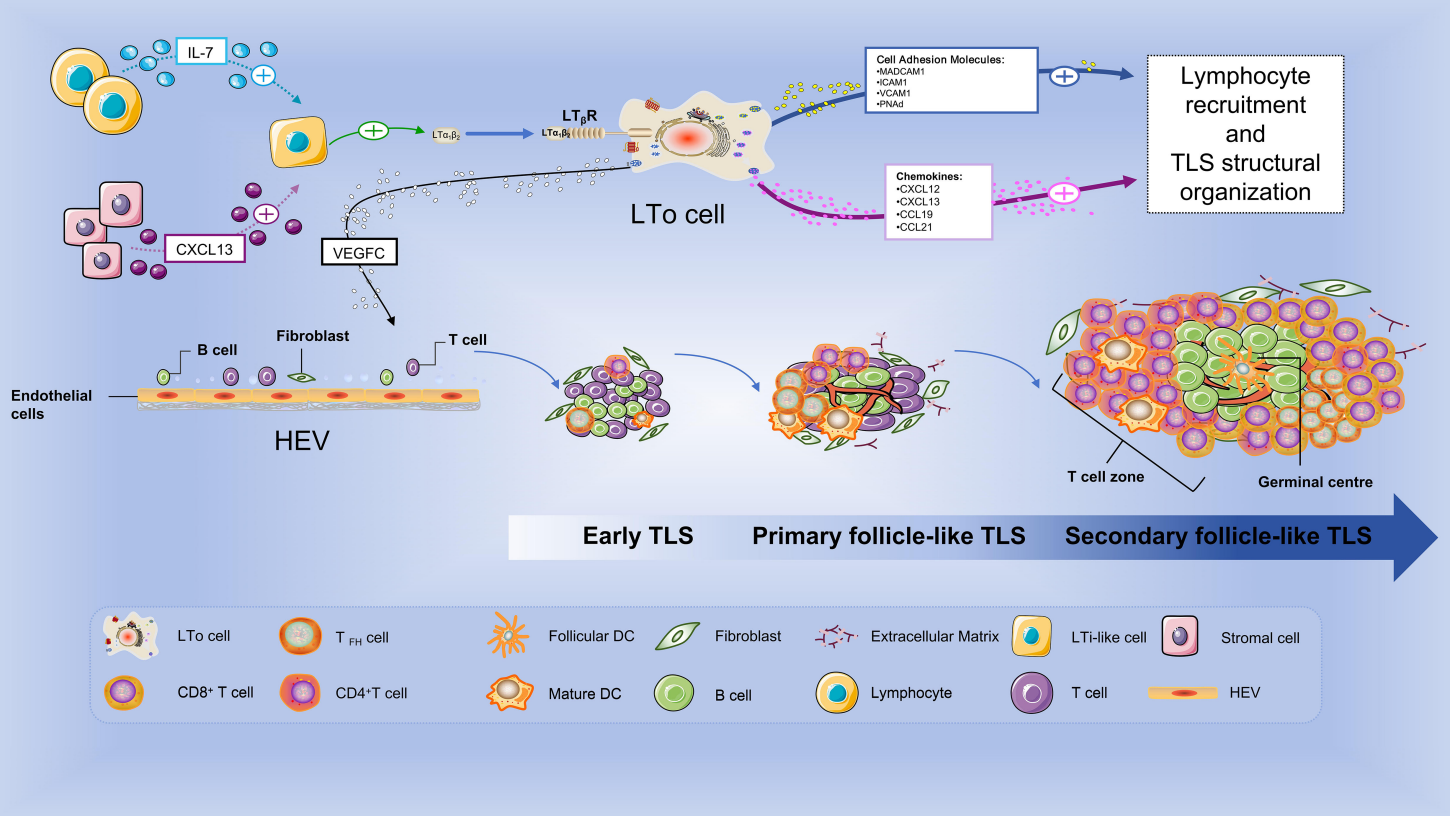
Formation process of TLS in renal cell carcinoma. The formation of tertiary lymphoid structures (TLS) in renal cell carcinoma (RCC) undergoes three main developmental stages: early TLS (E-TLS), primary follicle-like TLS (PFL-TLS), and secondary follicle-like TLS (SFL-TLS). Initially, lymphocytes and stromal cells release interleukin-7 and CXC chemokine ligand 13 (CXCL13) to recruit lymphoid tissue-inducing (LTi)-like cells (where Th17 cells, type 3 innate lymphoid cells (NCR+ILC3), M1 macrophages, CD8+ T cells, NK cells, and B cells can substitute for LTi cells under pathological conditions). The interaction between LTi-like cells and lymphoid tissue-organizing (LTo) cells (i.e., activated stromal cells) generates vascular endothelial growth factor C (VEGFC), adhesion molecules, and chemokines, which attract T cells and B cells to form E-TLS. Subsequently, B cells and T cells gradually compartmentalize to form PFL-TLS, although without germinal centers (GCs). Finally, the follicular dendritic cell (FDC) network emerges, the GC develops, and B cells mature to produce antibodies, ultimately forming SFL-TLS.

The process of TLS formation can be summarized as follows (**Figure 2**): Under inflammatory conditions, leukocytes release interleukin-13 (IL-13), IL-17, and IL-22, which induce the activation of stromal cells adjacent to sites of inflammation. Activated stromal cells or lymphocytes locally produce CXC-chemokine ligand 13 (CXCL13), IL-7, and vascular endothelial growth factor (VEGF)-A (28), recruiting lymphoid tissue-inducing (LTi)-like cells. LTi-like cells activate stromal cells expressing lymphotoxin-β receptor (LTβR) through secretion of lymphotoxin α1β2 (LTα1β2) or LIGHT (also known as TNFSF14, tumor necrosis factor superfamily member 14). These activated stromal cells are termed lymphoid tissue organizer (LTo) cells (29). Activated stromal cells secrete vascular endothelial growth factor C (VEGFC), promoting the development of high endothelial venules (HEVs), and produce adhesion molecules (MADCAM1, ICAM1, VCAM1, PNAd) and chemokines (CXCL12, CXCL13, CCL19, and CCL21), which recruit T and B cells via HEVs. Together with abundant fibroblasts, these components form E-TLS (30). LTi cells are a type of hematopoietic-derived cells present in both embryonic and adult stages, serving as key inducer cells that initiate and drive lymphoid tissue organogenesis (31). During the embryonic period, LTi cells originate from common lymphoid progenitors in the fetal liver (27), while in the formation of TLS in adult tissues (such as tumor or chronic inflammation sites), they are referred to as LTi-like cells (e.g., T helper 17 (Th17) cells, NCR+ ILC3s (type 3 innate lymphoid cells), M1 macrophages, CD8+ T cells, NK cells, and B cells, which can substitute for LTi cells to initiate TLS formation under pathological conditions) (30). LTo cells are a type of non-hematopoietic stromal cells, primarily mesenchymal stromal cells, which provide the structural scaffold and microenvironmental signals for lymphoid tissue formation. Based on their origin and function, LTo cells can be classified into different subsets: lymphatic endothelial LTo cells, mesenchymal LTo cells, and blood endothelial LTo cells (31, 32). During embryogenesis or the early postnatal period, LTo cells participate in the formation of SLOs; in adult vascularized tissues, they are involved in TLS formation, a process triggered by inflammatory reactions associated with various diseases (autoimmune diseases, infections, and cancer) or clinical contexts (transplantation) (29). LTi and LTo cells, through a cytokine-receptor interaction network (centered on the LTα1β2-LTβR axis), collectively constitute the cellular basis for the formation of both SLOs and TLSs. Their characteristics, origins, and interaction mechanisms are highly similar between embryonic development and adult pathological states, yet exhibit context-dependent cellular substitution and signaling pathway emphasis. After the formation of E-TLS, CXCL13 attracts B cells to establish lymphoid follicles, while CCL19 and CCL21 recruit T cells and DCs (33), thereby promoting compartmentalization of B and T cell zones and leading to the formation of PFL-TLS. PFL-TLS further develops into organized B and T cell zones with the emergence of FDC networks. Germinal centers begin to form, where B cells undergo maturation and antibody production, ultimately giving rise to SFL-TLS.

As a malignant tumor, RCC may influence the formation and maturation of TLS through its distinct biological pathways. Loss-of-function mutations in the *von Hippel-Lindau (VHL)* tumor suppressor gene are a hallmark of clear cell RCC (ccRCC) (34). When *VHL* is mutated or deleted, hypoxia-inducible factor-α (HIF-α) fails to undergo proteasomal degradation, resulting in the constitutive activation of downstream target genes, such as vascular endothelial growth factor (VEGF). VEGF not only promotes tumor angiogenesis but also facilitates the development of high endothelial venules (HEVs), thereby driving the formation of TLS. A recent study revealed that a subset of *VHL*-mutant ccRCC exhibits hyperactivation of the cell cycle and nuclear factor-κB (NF-κB) pathways, rather than the canonical HIF signaling pathway. Notably, these tumors are characterized by an inflamed tumor microenvironment enriched with TLS and demonstrate heightened sensitivity to immunotherapy (35). These findings suggest that NF-κB hyperactivation may promote TLS neogenesis, though the precise mechanisms remain to be elucidated. Emerging evidence indicates that within APC (antigen-presenting cell)-enriched niches of RCC, tumor-infiltrating CD8^+^ T cells can be stratified into terminally differentiated and stem-like subsets. Depletion of APC-rich regions correlates with tumor progression, implying a potential role for these niches in TLS formation during RCC pathogenesis (36).

In addition to RCC-specific signaling pathways, certain ubiquitous pathways may also promote TLS formation in RCC. The lymphocyte homing axis—including the CXCL13-CXCR5 and CCL19/CCL21-CCR7 axes—plays a pivotal role in lymphocyte aggregation. Specifically, the CXCL13-CXCR5 axis facilitates B-cell migration, while the CCL19/CCL21-CCR7 axis guides T-cell and dendritic cell (DC) trafficking. This migratory process primarily relies on high endothelial venules (HEVs) and their secreted adhesion molecules and chemokines, encompassing four critical steps: rolling, adhesion to HEVs, intraluminal crawling, and transendothelial migration (23). Notably, HEVs serve as a structural and functional bridge during this process, providing a dedicated pathway for lymphocyte recruitment while preventing direct contact between lymphocytes and tumor cells that might prematurely trigger anti-tumor immunity. This spatial segregation ensures targeted lymphocyte homing to specific niches, priming the microenvironment for subsequent TLS assembly. The role of positive feedback loops in TLS formation warrants emphasis (16, 22). In SLO, lymphocyte-derived chemokines establish self-amplifying circuits that drive the development of SLO. Analogous mechanisms likely operate in TLS. For instance, LTα1β2 binding to LTβR upregulates genes encoding lymphoid chemokines and HEV-specific factors. The resulting secretion of CCL19, CCL21, and CXCL13 further enhances lymphocyte recruitment. As lymphocytes accumulate, they amplify production of TNF-LT family cytokines and chemokines, creating a feedforward loop that sustains TLS neogenesis.

Current understanding of the cellular and molecular processes driving TLS formation is primarily derived from models of autoimmune diseases and chronic infections (18). In the context of cancer, however, inflammatory signals may not be the sole drivers of TLS induction and maintenance. Persistent tumor antigen stimulation has been proposed as another critical factor sustaining TLS development and function (29). Furthermore, the microbiota within the tumor microenvironment can promote TLS neogenesis. For instance, *Helicobacter hepaticus (Hhep)*-colonized mice exhibit increased TLS density in both peritumoral and intratumoral regions, which enhances TLS formation and functionality while potentiating anti-tumor immunity (37). These findings provide novel insights into the regulatory mechanisms underlying TLS assembly.

## 3 Classification and evaluation of TLS in RCC

### 3.1 Classification of TLS in RCC

#### 3.1.1 Classification based on TLS histopathological features

**Figure 3:**
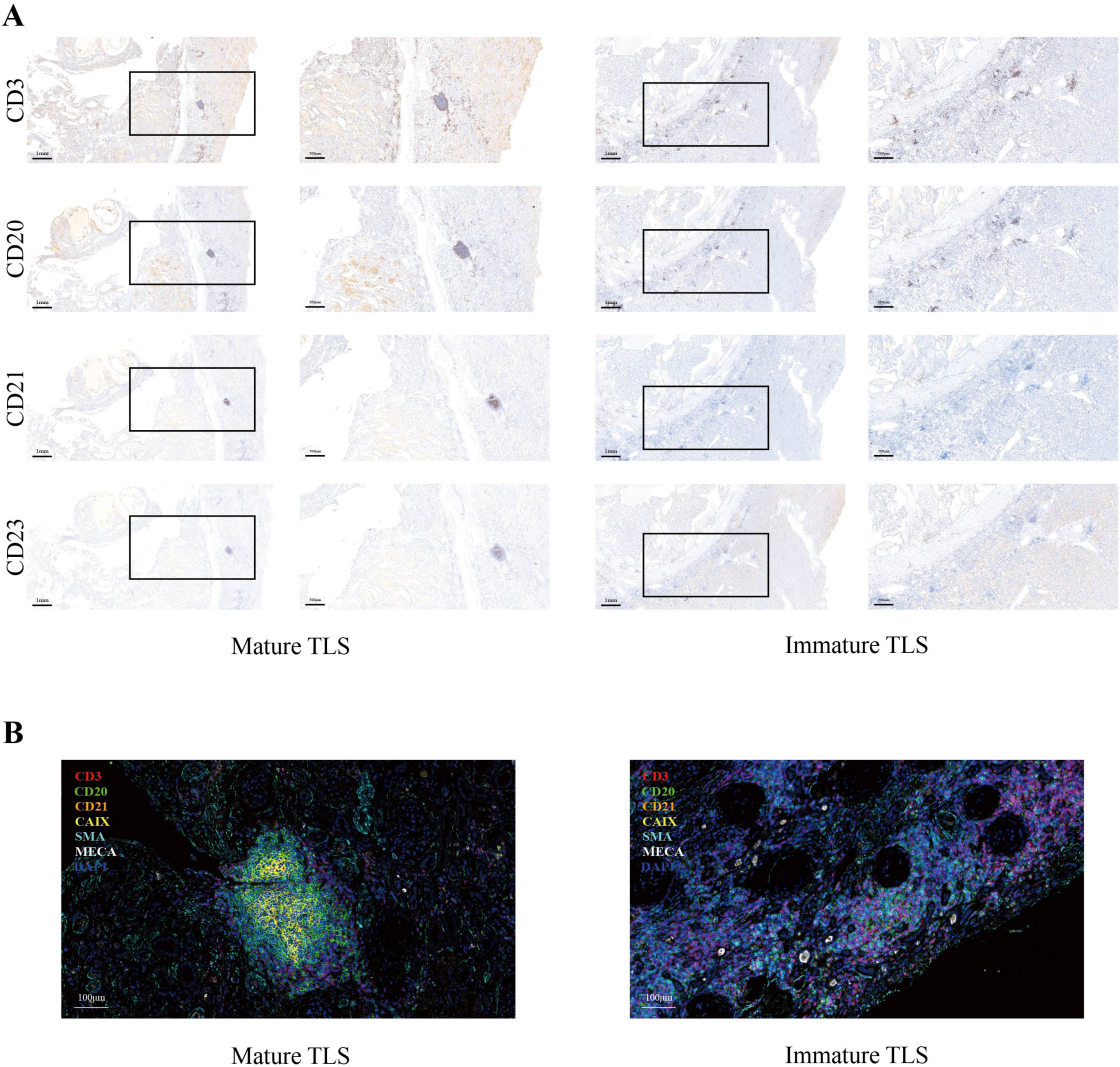
Heterogeneity in TLS maturation stages in renal cell carcinoma. **(A)** Immunohistochemical (IHC) assessment of tertiary lymphoid structures (TLS) maturation in serial sections. Sequential staining for CD3+ T cells, CD20+ B cells, CD21+ follicular dendritic cells (FDCs), and CD23+ germinal center (GC) cells was performed to classify TLS into distinct maturation stages. **(B)** Multiplex immunofluorescence (mIF) analysis of TLS maturation. Tissue sections were co-stained with a 7-marker panel (CD3, CD20, CD21, CAIX, SMA, MECA-79, and DAPI) to spatially resolve immune and stromal components within TLS at different maturation stages.

Based on histopathological features, TLS in RCC can be classified into mature and immature TLS. Mature TLS exhibit distinct yet adjacent T-cell and B-cell zones, active GCs, HEVs, and activated FDCs (CD21^+^CD23^+^) within B-cell areas (**Figure 3**; ref (24)). Current studies categorize mature TLS as encompassing both PFL-TLS and SFL-TLS (38). However, PFL-TLS contain immature FDCs and lack observable GCs. In one RCC study, PFL-TLS was defined as a transitional phase of TLS maturation (20), while another RCC study classified both E-TLS and PFL-TLS within the immature TLS group (39). Thus, whether PFL-TLS qualifies as mature TLS remains a matter of debate. Immature TLS display lymphoid aggregates of T and B cells but lack GCs and FDCs. Furthermore, a TLS maturity scoring system has been proposed for RCC, with samples dichotomized into mature and immature TLS groups based on median scores (40). In other malignancies such as hepatocellular carcinoma (HCC), TLS are stratified by maturity into aggregates (Agg), primary follicles (FL-I), and secondary follicles (FL-II) (41). Mature TLS generally correlate with favorable oncologic outcomes. A multicenter cohort study (n=720) employing Kaplan-Meier and Cox regression analyses demonstrated that mature TLS is associated with favorable prognosis in RCC. Using a median maturity score of 1.8 as the cutoff, the mature TLS group exhibited superior overall survival (OS) (p=0.005). The key effect size, the hazard ratio (HR) for mature versus immature TLS in the multivariate Cox regression, was 0.425 (95% CI: 0.110–1.633). Although this did not reach statistical significance, likely due to the limited sample size, it suggests a protective effect. Furthermore, in the treatment cohort, patients with mature TLS showed a higher treatment response rate (p=0.02) and longer progression-free survival (PFS) (p=0.002), further supporting its prognostic value (40). Conversely, immature TLS are frequently associated with adverse prognosis. For instance, their presence in premalignant lesions of HCC and colorectal cancer (CRC) correlates with elevated cancer recurrence rates (41, 42).

#### 3.1.2 Classification based on TLS spatial distribution

**Figure 4:**
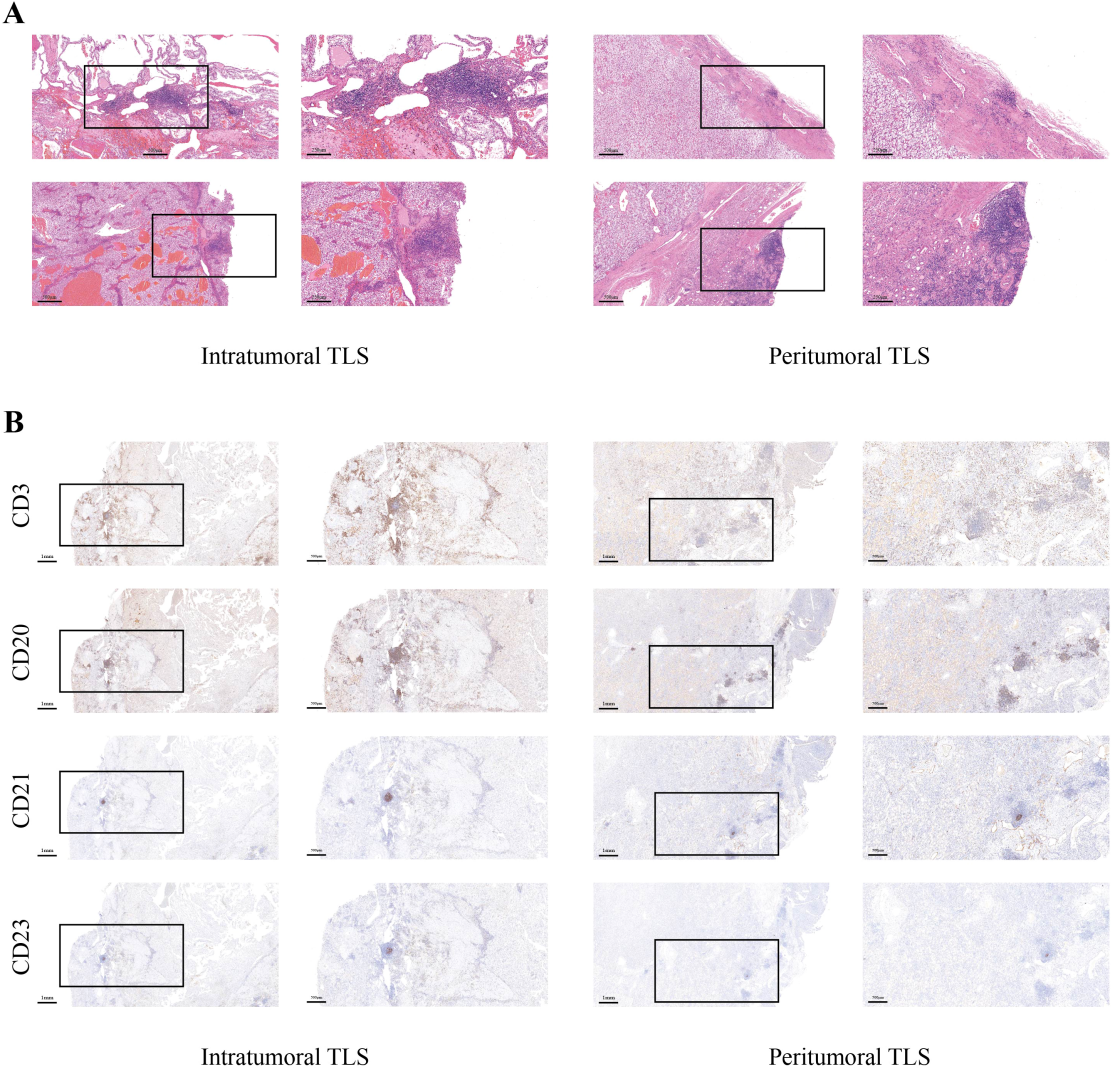
Spatial heterogeneity of TLS localization in renal cell carcinoma.**(A)** Representative H&E-stained serial sections distinguishing intratumoral versus peritumoral TLS. **(B)** Consecutive sections were subjected to immunohistochemical (IHC) staining for CD3^+^ T cells, CD20^+^ B cells, CD21^+^ follicular dendritic cells, and CD23^+^ germinal center cells.

Based on the spatial relationship between TLS and tumor tissue, RCC TLS can be classified into intratumoral, invasive margin (IM), and peritumoral categories (**Figure 4**). The IM is defined as fibrous tissue located within 1 mm of tumor cells (43). However, the exact cutoff value remains inconsistent across studies. Alternative classification systems categorize TLS as tumor-proximal (located within the IM) or tumor-distal (situated in normal tissue more than 10 mm from the IM) (20). Emerging evidence suggests distinct prognostic implications for different TLS spatial distributions in RCC. Using the intratumoral/IM/peritumoral classification, intratumoral TLS in RCC predominantly exhibit SFL-TLS characteristics and correlate with favorable outcomes, whereas peritumoral TLS are primarily E-TLS associated with poor prognosis (39). When applying the tumor-proximal/distal classification, Meylan et al. demonstrated that tumor-proximal TLS predict favorable prognosis (17), while Masuda et al. reported contradictory findings linking tumor-distal TLS with adverse outcomes (44). In the study by Wenhao Xu et al., which utilized a multicenter cohort and survival analysis, it was revealed that the spatial distribution of TLS significantly influences RCC prognosis, with effect sizes of clinical relevance. In the training set (n=290), tumor-proximal TLS were associated with longer PFS (HR = 0.387, 95% CI not explicitly stated but p=0.002) and OS (HR = 0.456, p<0.001); whereas tumor-distal TLS predicted poor outcomes (PFS: HR = 1.486, p=0.020; OS: HR = 1.444, p=0.021). This consistent trend was replicated in an external validation set (n=105) (proximal PFS HR = 0.167, OS HR = 0.263; distal PFS HR = 1.993, OS HR = 2.024). Consequently, they concluded that tumor-proximal TLS indicate a favorable prognosis, while tumor-distal TLS suggest a poorer prognosis. Interestingly, Wenhao Xu et al. observed SFL-TLS predominance in tumor-proximal regions versus E-TLS features in the distal areas, with tumor-distal TLS-positive specimens showing significantly increased PD-L1^+^ tumor-associated macrophages and regulatory T cell infiltration, suggesting an immunosuppressive microenvironment (20). These spatial distribution patterns extend beyond RCC – in intrahepatic cholangiocarcinoma (iCCA), intratumoral TLS predict favorable prognosis while peritumoral TLS correlate with poor outcomes (45). Collectively, TLS spatial heterogeneity in RCC demonstrates significant prognostic relevance, underscoring its potential for refining risk stratification and guiding immunotherapeutic strategies.

#### 3.1.3 Other classification methodologies

The aforementioned two classification systems represent the predominant methodologies for TLS categorization in RCC. Additional studies have classified TLS based on its presence or absence (TLS-positive vs. TLS-negative), although no standardized definition for TLS positivity currently exists. Some investigations define TLS-positivity as the presence of at least one intratumoral TLS (45), while others require a minimum of two lymphoid aggregates containing both CD20^+^ B cells and CD3^+^ T cells (46). Quantitative criteria have also been proposed, with certain studies defining TLS-positivity as lymphoid aggregates in tumor or invasive margin regions containing more than 50 lymphocytes (43). Alternative approaches incorporate spatial parameters, classifying TLS-positive cases based on CD3^+^ T cell aggregates with CD20^+^ B cells occupying an area of more than 7,000 μm² and containing at least 100 cells (17). Furthermore, certain studies have utilized the density of TLS as a classification criterion, categorizing TLS into high-density and low-density subtypes (45, 47). However, there is no consensus yet on issues such as what constitutes “high” density or “low” density, and how to determine the critical value for distinguishing between high and low density. Therefore, it is difficult to use TLS density as a classification standard for TLS.

From a clinical perspective, TLS classification in RCC may assist clinicians in prognostic evaluation and therapeutic decision-making. Patients with high-density, mature TLS typically demonstrate favorable prognoses and a lower risk, whereas those lacking TLS or exhibiting predominantly immature TLS face an elevated risk of recurrence. Furthermore, TLS maturation status correlates with response to ICIs, with mature TLS potentially associated with improved therapeutic outcomes following ICI-based combination therapy (48). Consequently, future clinical applications may integrate comprehensive assessments of TLS composition, maturation status, spatial distribution, and density to stratify patients into high-risk and low-risk groups. This classification framework provides a rationale for personalized treatment strategies and underscores the critical role of TLS heterogeneity in RCC immunotherapy.

### 3.2 Quantitative assessment techniques for TLS in RCC

Accurate assessment of TLS maturity, spatial distribution, quantity, and density is critical for investigating the prognostic relevance of TLS in RCC. Currently, there is no standardized evaluation system for TLS. While the inclusion/exclusion criteria and baseline characteristics for patients may vary among researchers based on their research objectives, the quantitative assessment techniques for TLS show minimal variation. With rapid advancements in artificial intelligence (AI) and multi-omics technologies, future studies are expected to establish objective, reproducible, and standardized TLS assessment frameworks, thereby enhancing the representativeness and generalizability of research findings. Such progress will strengthen the evidentiary basis for utilizing TLS as both a prognostic biomarker and therapeutic target in RCC.

#### 3.2.1 Inclusion and exclusion criteria and baseline characteristics

Current research on TLS in renal cell carcinoma predominantly consists of retrospective studies. Common patient inclusion criteria are: (1) patients with histologically confirmed RCC; (2) availability of tumor tissue specimens suitable for TLS evaluation (typically surgical resection specimens); (3) patients who have not received preoperative neoadjuvant therapies such as chemotherapy, targeted therapy, radiotherapy, or immunotherapy; (4) complete clinicopathological data and follow-up information. Exclusion criteria are largely consistent across most studies, typically including: (1) pathologically confirmed non-renal cell carcinoma; (2) incomplete electronic medical records or follow-up data; (3) tumor specimens lacking paired adjacent normal tissue (20, 40). However, inclusion and exclusion criteria may vary based on specific research objectives. For instance, cohorts specifically investigating the efficacy of ICIs would need to include patients treated with ICIs or combination therapy with tyrosine kinase inhibitors (TKIs), requiring measurable lesions and sufficient life expectancy (35, 39). Genomic studies utilizing public databases (e.g., TCGA) would exclude patients lacking survival information or genomic data (49). Baseline characteristics of studies generally encompass demographics (e.g., sex, age), pathological features (e.g., pathological stage, tumor grade), TLS detection rate, TLS-related characteristics such as composition, maturity, spatial distribution, and density, and associations with treatment response. Considerable variation exists in the baseline characteristics across different studies; consequently, the representativeness and generalizability of the corresponding findings warrant further investigation.

#### 3.2.2 Histological detection

Histological detection currently represents one of the most widely utilized techniques for TLS assessment, encompassing hematoxylin and eosin (HE) staining, immunohistochemistry (IHC), multiplex immunohistochemistry (mIHC), immunofluorescence (IF), and multiplex immunofluorescence (mIF) (**Table 1**). Additional methodologies include hematoxylin ^+^ 3,3’-diaminobenzidine (H-DAB) staining, hematoxylin-diaminobenzidine ^+^ alkaline phosphatase (H-DAB ^+^ AP) staining, and flow cytometry (FCM) (51). Each of these techniques presents distinct advantages and limitations. HE staining constitutes the most fundamental histological analytical approach, offering simplicity of execution and low cost. It enables preliminary identification of TLS presence through evaluation of gross morphological and architectural features in tissue sections. However, it lacks the resolution for detailed analysis of TLS histological characteristics. Recent studies have employed whole-slide imaging (WSI) of conventional HE-stained sections to quantitatively assess TLS (41, 52), though manual pathologist evaluation remains subject to substantial interobserver variability and limited reproducibility. To address this, automated computational pipelines have been developed for standardized TLS quantification (53), demonstrating both efficiency and accuracy (54). Comparative analyses reveal strong concordance between automated TLS enumeration and manual pathologist-based TLS counts derived from HE-stained WSI (55). IHC facilitates identification of immune cell subsets within TLS through targeted antigen labeling (e.g., CD20, CD3), yet it cannot reliably evaluate spatial relationships between multiple markers. It permits the simultaneous detection of 2–3 biomarkers, enabling the investigation of cellular interactions within TLS, but suffers from fluorescence signal decay and technical complexity. mIHC and mIF technologies leverage high-throughput capabilities to enable *in situ* profiling of multiple biomarkers across entire tissue sections within short timeframes, thereby characterizing cellular phenotypes, abundance, functional states, and intercellular relationships. While these advanced techniques provide comprehensive morphological and compositional data regarding TLS, they entail elevated technical costs, operational complexity, and specialized hardware/analytical expertise requirements (56). It is noteworthy that in histological detection, for histological analyses, 10% neutral-buffered formalin fixation with paraffin embedding (FFPE) remains the predominant method, although methanol fixation has been employed in selected studies (17). Alternatively, 4% paraformaldehyde demonstrates superior fixation homogeneity with enhanced preservation of cellular and architectural morphology, particularly advantageous for TLS structural analysis.

**Table 1:** TLSs in renal cell carcinoma: detection, marker, maturity, location, prognosis, immunotherapy response.

Disease	Detection	Marker (IHC)	Maturity	Location	Prognosis	Immunotherapy response	Ref
ccRCC	H&E	CD3	Mature	Tumor-proximal	Positive	NM	(20)
	IHC	CD20	Immature	Tumor-distal	Negative	NM	
	mIHC	CD21					
	mIF	CD23					
ccRCC	H&E	CD3	Mature	Intratumoral	Positive	Positive	(39)
	IHC	CD20	Immature	Peritumoral	Negative	Negative	
	mIHC	CD21					
	mIF	CD23					
	ST						
ccRCC	H&E	CD3	Mature	NM	Positive	Positive	(40)
	IHC	CD20					
	mIF	CD21					
ccRCC	H&E	Bcl6	Immature	NM	Negative	Negative	(44)
	IHC	CD3					
	DNA-seq	CD10					
		CD20					
		CD21					
RMC	H&E	SMARCB1	Mature	NM	Positive	Positive	(48)
	IHC	CD8					
	mIF	PD-L1					
	RNA-seq						
	WES						
RCC	H&E	NM	NM	NM	Positive (high-TLS tumor)	Positive (high-TLS tumor)	(50)
	mIF						
	RNA-seq						
	WES						

ccRCC, clear cell renal cell carcinoma; RMC, renal medullary carcinoma; H&E, hematoxylin and eosin; IHC, immunohistochemistry; mIHC, multiplex immunohistochemistry; mIF, multiplex immunofluorescence; ST, spatial transcriptomics; NM, not mentioned; DNA-seq, DNA sequencing; RNA-seq, RNA sequencing; WES, whole-exome sequencing; Ref., Reference.

#### 3.2.3 Transcriptomic analysis techniques

Transcriptomic analysis techniques are playing an increasingly pivotal role in TLS evaluation. These methodologies—including RNA sequencing (RNA-seq), single-cell RNA sequencing (scRNA-seq), and spatial transcriptomics (ST)—enable in-depth molecular characterization of TLS composition, functionality, and disease-associated dynamics. For instance, integrated scRNA-seq and spatially enhanced resolution omics sequencing (Stereo-seq) revealed preferential clustering of IgG^+^ plasma cells (PCs) near TLS regions. At the same time, IgA^+^ PCs were dispersed in non-TLS areas within colorectal cancer liver metastases (57). While RNA-seq and scRNA-seq lack spatial context, spatial resolved transcriptomics (SRT) uniquely preserves cellular spatial architecture while providing gene expression profiles (58). This capability has positioned SRT as a transformative tool in RCC-TLS research. Meylan et al. performed spatial transcriptomic analysis of RCC specimens, demonstrating dense plasma cell infiltration within mature TLS and identifying extra-TLS plasma cells in close spatial association with CXCL12^+^ fibroblasts. These findings support a mechanistic model wherein IgG^+^ and IgA^+^ plasma cells migrate along CXCL12-expressing fibroblastic networks to disseminate throughout tumor tissue (17). As SRT methodologies mature and become more accessible, they will further elucidate the cellular architecture and spatial distribution of TLS. Collectively, these transcriptomic approaches have expanded TLS evaluation beyond morphological assessment to molecular profiling, providing a powerful tool for in-depth exploration of the molecular mechanism of TLS in RCC.

#### 3.2.4 Imaging histology assessment techniques

Imaging histology techniques have provided novel insights into TLS research, particularly in non-invasive evaluation. For instance, preoperative contrast-enhanced CT imaging features can predict intra-tumoral TLS in HCC (59). Compared to clinical models, radiomics models demonstrated superior discriminative performance in cohorts, suggesting that radiomic features serve as non-invasive biomarkers for distinguishing TLS in patients with invasive pulmonary adenocarcinoma (IA) (60). However, one study utilizing preoperative CT radiomics to predict TLS status and recurrence-free survival (RFS) in intrahepatic cholangiocarcinoma (ICC) patients found that a combined model integrating clinical and radiomics features exhibited the best predictive performance (61). Compared to CT, MRI offers superior soft-tissue resolution and avoids radiation exposure. Shichao Long et al. developed an enhanced contrast-based MRI radiomics model to non-invasively predict high peritumoral TLS (pTLS) density in HCC, which was associated with favorable prognosis and response to immune checkpoint inhibitor (ICI)-based combination therapy (62). Additionally, Shichao Long et al. constructed a transfer learning radiomics (TLR) model based on multiphase MRI, enabling non-invasive prediction of intra-tumoral TLS (iTLS), thereby providing a basis for risk stratification in clinical oncology (63). In summary, imaging histology, a non-invasive approach, demonstrates significant potential in assessing TLS in RCC. Future studies may develop clinical models, radiomics models, or combined models based on CT or MRI for the evaluation of TLS in RCC. However, this technology has limitations, including the need for further investigation into model interpretability. Additionally, the use of retrospective cohorts in current studies may introduce selection bias, necessitating validation through prospective research to confirm model applicability.

## 4 Dual-functional roles of TLS in RCC: anti-tumor activity versus immunosuppression

As previously discussed, the composition, maturation status, spatial distribution, and density of TLS have a significant influence on cancer prognosis. In most malignancies (e.g., lung cancer (64, 65), ovarian cancer (66), colorectal cancer (42, 57), and bladder cancer (67, 68)), TLS are associated with improved clinical outcomes and enhanced responses to immunotherapy. However, in RCC, the relationship between TLS and patient prognosis or immunotherapy response demonstrates notable heterogeneity, with some studies even reporting associations with poorer outcomes and diminished therapeutic responses. Therefore, it is imperative to discuss the anti-tumor effects and immunosuppressive mechanisms of TLS in RCC.

### 4.1 Anti-tumor effects and mechanisms of TLS in RCC

Given that TLS share compositional, structural, and functional similarities with SLO, they can functionally substitute for SLO in generating specific adaptive anti-tumor responses, thereby effectively inhibiting tumor growth and metastasis. Based on current research on TLS in cancer, the potential mechanisms underlying the anti-tumor effects of TLS in RCC may be elucidated as follows:

B cell-mediated anti-tumor immunity. Mature TLS exhibit active germinal center (GC) reactions, wherein B cells undergo maturation, selection, proliferation, somatic hypermutation, affinity maturation, and immunoglobulin class switching, ultimately differentiating into plasma cells secreting IgG or IgA. Tumor-infiltrating plasma cells generated *in situ* within TLS can produce antibodies targeting specific tumor-associated antigens, thereby exerting anti-tumor effects (69, 70). For instance, in RCC patients, a robust B cell response was observed within TLS and adjacent regions, with IgG- and IgA-positive plasma cells disseminating along CXCL12-positive fibroblast trajectories in the tumor microenvironment (17). Tumor-specific antibodies from plasma cells activate multiple effector mechanisms: (1) complement pathway activation via C1q binding (71), (2) Fcγ receptor (FcγR)-mediated phagocytosis by NK cells or macrophages (72), and (3) complement-dependent cytotoxicity (CDC) or antibody-dependent cellular cytotoxicity (ADCC) – evidenced by IgG-coated tumor cells in RCC (73). B cells also contribute to anti-tumor immunity by functioning as antigen-presenting cells (APCs). Within TLS, B cells process and present antigenic peptides to CD4^+^ T cells via the major histocompatibility complex (MHC) class II system and can cross-present peptide-MHC class I complexes to CD8^+^ T cells (74), thereby enhancing anti-tumor responses. Furthermore, studies of RCC and melanoma patients treated with anti-PD-1 monotherapy or combined anti-CTLA-4 (cytotoxic T-lymphocyte antigen-4) therapy revealed that responders exhibited upregulated B cell-related signatures and higher densities of B cells and TLS in tumors and peripheral blood compared to non-responders (12). These findings underscore the critical role of TLS-resident B cells in immunotherapy response.

T cell-mediated anti-tumor immunity. Studies in non-obese diabetic (NOD) mouse models demonstrate that LIGHT-induced TLS recruit naive T cells to pancreatic islets and promote their *in situ* proliferation (75), suggesting potential analogous mechanisms for naive T cell activation within RCC-associated TLS. As most tumor-associated TLS lack a defined capsule, immune cells within TLS directly interact with tumor cells, enabling APCs to activate T cells more efficiently and potentiate their anti-tumor effects. In studies examining the impact of immune infiltration signatures in TLS from non-small cell lung cancer (NSCLC) patients, the density of mature DCs within TLS was correlated with the overexpression of genes associated with T cell activation, Th1 polarization, and cytotoxic effector functions, thereby shaping a distinct intratumoral immune milieu that enhances T cell-mediated anti-tumor immunity (76). These findings underscore the role of TLS as pivotal activation hubs for T cells in anti-tumor immune responses (77). However, it remains unresolved whether TLS primarily participate in the activation of naive T cells or the reactivation of effector T cells.

Interplay Between T Cells and B Cells in TLS. Follicular helper T cells (Tfh cells), predominantly localized within and adjacent to B cell zones, facilitate B cell activation, proliferation, and differentiation into memory B cells and antibody-secreting plasma cells (29). The IgG1 antibodies produced by plasma cells form immune complexes with tumor antigens, which are subsequently internalized with high efficiency by DCs via activated Fc γ receptors (FcγRs), thereby enhancing antigen presentation to T cells and amplifying T cell responses (78). Furthermore, clonotypic profiling of T cells across distinct tissue compartments in NSCLC patients revealed that elevated CD4^+^ T cell clonality within tumors correlated with increased B cell density in TLS (79), suggesting that tumor-infiltrating T cell clonal expansion may be linked to TLS-resident B cells—a phenomenon potentially attributable to B cell-mediated antigen presentation. These findings support the hypothesis that in RCC, TLS-derived T and B cells may engage in specialized cross-talk mechanisms to mutually potentiate their respective anti-tumor functions, synergistically enhancing both T cell- and B cell-mediated immune responses against tumors.

RCC-associated TLS may further enhance anti-tumor immunity by modulating the cytokine network within the TME, thereby promoting sustained activation and proliferation of immune cells (80, 81). B cells within TLS secrete various cytokines (including TNF, IL-2, IL-6, and IFN-γ) that activate and recruit other immune cells, particularly T cells exhibiting high infiltration density in the peri-TLS regions (12). Concurrently, the positive feedback mechanism described during TLS formation promotes TLS maturation through the activity of cytokines and chemokines. Given that mature TLS generally correlate with favorable prognosis in cancer patients, this process appears to augment anti-tumor immune capacity. In summary, the anti-tumor effects mediated by RCC-associated TLS likely involve multiple immune cell types, cytokines, and signaling pathways. However, the unique TME characteristics of RCC and the complex cytokine regulatory networks within TLS may render the immunomodulatory processes more intricate than those of other cancer types. Consequently, future investigations should focus on elucidating the molecular mechanisms underlying TLS-mediated anti-tumor immunity in RCC.

### 4.2 Immunosuppressive effects and mechanisms of TLS in RCC

Although TLS play a crucial role in anti-tumor immunity, they may also contribute to immunosuppression under certain circumstances, thereby promoting tumor progression. This phenomenon is particularly evident in RCC, where several studies have reported associations between TLS and poor patient prognosis. The potential immunosuppressive mechanisms mediated by TLS in RCC may include the following aspects:

Recent studies on TLS in RCC have revealed that TLS heterogeneity may be a critical determinant of patient prognosis. The composition of RCC-associated TLS varies significantly depending on their maturation status, spatial distribution, and density. Consequently, when TLS are found to correlate with poor clinical outcomes, detailed characterization of their cellular composition may provide insights into their immunosuppressive mechanisms. A retrospective study integrating intratumoral TLS and tumor-infiltrating lymphocytes (TILs) in RCC demonstrated that mature, high-density TLS were associated with favorable prognosis. In contrast, high TIL proportions were associated with adverse outcomes (40). Notably, the proliferative activity of TILs showed more substantial predictive value for survival than their absolute infiltration density (82). These immunosuppressive TILs exhibited an exhausted phenotype characterized by elevated expression of PD-1, T-cell immunoglobulin and mucin-domain containing-3 (TIM3), inducible T-cell costimulator (ICOS), and tumor necrosis factor receptor superfamily member 9 (TNFRSF9) (83). Masuda et al. reported significantly higher frequencies of PD-1^+^ cell infiltration in RCC TLS compared to other urological malignancies (44), indicating pronounced T-cell exhaustion within RCC TLS. While PD-1 is absent on quiescent T cells, its expression on activated effector T cells leads to lymphocyte suppression upon ligand binding (84), thereby attenuating anti-tumor immunity. The absence of encapsulating structures in RCC TLS permits sustained lymphocyte-tumor antigen contact, which may impair CD8^+^ T-cell proliferation and cytotoxic function (85). However, emerging evidence suggests that TILs comprise both TCF1^+^ stem-like CD8^+^ T-cell populations and their terminally exhausted progeny, which express high levels of checkpoint molecules. The decline in T-cell responses may stem from inadequate stimulation of TCF1^+^ progenitor cells by APCs, resulting in continuous generation of exhausted CD8^+^ T cells that facilitate immune escape (36). Further investigations revealed that intratumoral infiltration of CXCL13^+^CD8^+^ T cells correlated with poorer clinical outcomes in RCC patients (86). Given the positive association between CXCL13^+^CD8^+^ T-cell density and TLS presence (87), it is plausible that RCC TLS may promote immunosuppression by increasing exhausted CD8^+^ T-cell infiltration among TILs, potentially through TLS heterogeneity-driven mechanisms. Additionally, RCC specimens with tumor-distal TLS exhibited elevated PD-L1^+^ tumor-associated macrophages and regulatory T-cell infiltration, which were associated with reduced progression-free survival (PFS) and OS (20). These findings suggest complex interplay between RCC TLS and TILs, warranting future investigations into the regulatory mechanisms underlying TLS composition, maturation status, spatial distribution, and density.

TLS may also remodel the renal carcinoma microenvironment through cytokine production, fostering an immunosuppressive milieu inducing to tumor progression. For instance, immune cells within TLS can alter the cellular composition of the TME via proinflammatory factor secretion, ultimately establishing an immunosuppressive state that enables tumor immune evasion (81, 88). Immature TLS-derived B cells generate inhibitory factors that suppress the activity of other immune cells (e.g., T cells), while insufficient antigen presentation further dampens local immune responses (89). During this process, B cell-secreted TGF-β drives macrophage polarization toward the M2 immunosuppressive phenotype (90, 91), thereby creating a permissive microenvironment for tumor growth and metastasis. In addition, B cells can secrete VEGF to promote the formation of tumor blood vessels, thereby facilitating tumor progression (92). Genomic analyses of RCC specimens with pathway interrogation revealed that alterations in the PI3K-mTOR pathway were prevalent in TLS-positive tumors, driving the excessive production of proinflammatory and proangiogenic factors (44). Concurrently, *VHL* gene mutations or deletions—a hallmark of ccRCC—induce aberrant cytokine production (93). These cytokine surges may establish an immunosuppressive TME (84), collectively facilitating tumorigenesis and progression.

Overall, in RCC, the anti-tumor and immunosuppressive effects of TLS rely on the delicate equilibrium of the density, phenotype, and activity of diverse cell types. Additionally, it is also influenced by the interaction between the unique tumor microenvironment of RCC and TLS. A thorough understanding of the dual mechanisms of TLS in anti-tumor and immunosuppressive effects in RCC will provide novel theoretical foundations and research directions for immunotherapy strategies in RCC.

## 5 Clinical translation of TLS in RCC: from prognostic biomarker to therapeutic target

### 5.1 Prognostic and therapeutic potential of TLS in RCC

TLS, functioning as “plastic immune organs,” demonstrate dual potential as both prognostic biomarkers and therapeutic targets in RCC. Unlike in other malignancies, TLS in RCC exhibit associations with dichotomous prognostic outcomes, rendering their mere presence inadequate for predicting patient prognosis. Current evidence suggests this paradox may stem from TLS heterogeneity. Specifically, tumor-proximal TLS correlate with favorable prognosis while tumor-distal TLS are predominantly associated with poorer outcomes in RCC patients, with additional variations observed in maturation status across different tumor locations (20). Notably, a subset of *VHL*-mutated ccRCC exhibits TLS-rich tumor microenvironments yet demonstrates unexpectedly poor prognosis (35). Intriguingly, these patients display heightened sensitivity to immunotherapy, suggesting that therapeutic intervention may modulate TLS characteristics, including cellular composition, maturation status, spatial distribution, and density. This observation highlights the potential of TLS as a therapeutic target for immunotherapy. While intratumoral TLS have demonstrated predictive value for ICI response in certain malignancies (13, 94, 95), research in RCC remains limited. Recent findings suggest that TLS presence serves as a necessary but insufficient condition for a favorable ICI response, as therapeutic efficacy may be compromised by elevated infiltration of exhausted CD8+ T cells in TLS-high tumors (50). Another comprehensive evaluation of TLS spatial distribution and maturation status in RCC revealed that intratumoral TLS and SFL-TLS significantly correlate with improved survival rates and objective response rates following ICI treatment (39). Furthermore, the prognostic value of TLS exhibits age-dependent variations among patients with RCC (40). In conclusion, the presence of TLS alone cannot reliably predict RCC prognosis, with TLS heterogeneity emerging as a critical determinant of clinical outcomes. A systematic investigation of TLS characteristics, including cellular composition, maturation stage, spatial architecture, and density, in relation to RCC prognosis is imperative for developing precise prognostic models and personalized therapeutic strategies.

### 5.2 TLS-targeted biotherapeutic strategies in RCC

Given the demonstrated dual potential of TLS as both prognostic biomarkers and therapeutic targets in RCC, coupled with their functional heterogeneity within the tumor microenvironment, where they can either promote anti-tumor immune responses or, in certain contexts, contribute to immunosuppression, precisely targeted modulation strategies for TLS hold significant clinical implications. The selective induction of anti-tumor TLS formation or suppression of TLS subtypes that facilitate immune evasion may provide novel therapeutic approaches to improve clinical outcomes for RCC patients.

#### 5.2.1 Therapeutic induction of TLS

Inducing the formation and maturation of TLS is a promising approach for immunotherapy. Current research primarily focuses on key biological events during TLS formation, including lymphocyte recruitment, regulation of the chemokine network, and modulation of the tumor microenvironment. Targeted interventions at these critical stages can promote TLS development and maturation while enhancing their anti-tumor immune capacity. For instance, in mouse lung adenocarcinoma models, Tregs were found to suppress anti-tumor responses within TLS, and Treg depletion significantly increased proliferation rates of DCs and T cells in TLS (96), which suggests that inhibiting immunosuppressive cell function and modifying the immunosuppressive microenvironment may facilitate TLS formation. More direct approaches are gaining attention, such as injecting stromal cells to substitute for LTo cell functions (97, 98). Enhancing interactions between LTo-like cells and LTi-like cells promotes the release of relevant cytokines and chemokines, thereby inducing the formation of TLS. Alternatively, intratumoral delivery of cytokines or chemokines, such as CCL19, CCL21, and CXCL13, can recruit and aggregate immune cells, leveraging positive feedback mechanisms to stimulate TLS development further. For example, in mouse pancreatic ductal adenocarcinoma (PDAC) models, intratumoral injection of lymphoid chemokines CXCL13 and CCL21 induced *in situ* TLS formation. Furthermore, combining systemic chemotherapy with intratumoral lymphoid chemokine administration altered immune cell infiltration, enhanced TLS induction, and improved the anti-tumor efficacy of chemotherapy (99). These findings support the feasibility of integrating such strategies with existing immunotherapies.

It is noteworthy that, as mentioned earlier, the VEGF pathway may be involved in TLS formation. Specifically, various isoforms of VEGF-A, through their unique structural characteristics and receptor interactions, can finely regulate angiogenesis in both physiological and pathological contexts, whereas VEGF-C plays a more complex role in cancer development, metastasis, and tumor-associated lymphangiogenesis (100). Therefore, how VEGF-A and VEGF-C participate in TLS formation and which one plays a dominant role require further in-depth investigation. Currently, several VEGF-targeted agents (such as bevacizumab and various VEGF-TKIs) have been approved and are widely used in RCC treatment, covering first-line, second-line, and later-line therapies (84). A study involving ccRCC patients found that the patient group characterized by an immune-”cold” and TLS-excluded microenvironment exhibited significantly higher sensitivity to VEGF-targeted drugs such as sunitinib, sorafenib, pazopanib, and axitinib compared to other groups. This finding suggests that a TLS-deficient or immune-”cold” tumor microenvironment may serve as a predictive biomarker for greater benefit from VEGF-targeted monotherapy in ccRCC patients (101). In contrast to monotherapy, when combined with ICIs, anti-VEGF therapy has demonstrated the capacity to remodel the tumor immune microenvironment and actively induce TLS formation. Preclinical and translational studies have confirmed that combining anti-PD-L1/PD-1 therapy with anti-angiogenic treatment can promote the transformation of tumor vasculature into HEVs, thereby inducing TLS formation (102). In a preclinical model of anaplastic thyroid cancer, the combination of anti-PD-1 therapy with famitinib, a multi-targeted tyrosine kinase inhibitor against VEGF/PDGF pathways, also effectively induced TLS formation within thyroid tumors (103). Interestingly, a study on HCC found that VEGF-C therapy could promote TLS formation by activating lymphangiogenesis and the CCL21/CCR7 axis, thereby enhancing the efficacy of anti-PD-1 immunotherapy. This provides some theoretical basis for understanding the mechanisms by which the VEGF pathway participates in TLS formation (104). However, it is regrettable that research on the impact of VEGF-targeted therapy on TLS in the field of RCC remains relatively limited to date.

Recent advances in oncolytic virus (OV) therapy have demonstrated significant potential for TLS induction, owing to their dual capacity as tumor-targeting vectors and activators of anti-tumor immunity (105). Engineered OVs can effectively deliver cytokines or chemokines to tumor sites, with studies showing that adenovirus serotype 5 (Ad5) expressing interleukin-15 (IL-15) promotes vascular normalization and generates CD45^+^ cell aggregates associated with PNAd^+^ HEVs, resembling TLS-like structures (106). Similarly, Ad5 vectors co-expressing tumor necrosis factor-α (TNF-α) and IL-2 have been shown to induce fully mature TLS formation in infected tumor models (107). Beyond their role as delivery vehicles, OVs can modulate molecular pathways critical for TLS development. In murine models, oncolytic herpes simplex virus-1 (oHSV) was found to activate the CXCL10/CXCR3 axis, triggering immune activation that culminates in TLS formation and consequent enhancement of immunotherapeutic efficacy (108).

Beyond OVs, emerging studies have explored alternative delivery platforms for TLS-inducing agents, including nanoparticles (109, 110), biological scaffolds (111), and hydrogels (112, 113), though their precise mechanisms in TLS induction require further elucidation. Notably, a hierarchical protein hydrogel encapsulating spleen-derived extracellular vesicles (sEVs) has been developed for the induction of TLS. Subcutaneous administration of this sEV-loaded hydrogel in murine models promoted angiogenesis, facilitated immune cell recruitment and aggregation, and ultimately induced mature TLS formation (114). Additionally, receptor agonists demonstrate TLS-inducing potential, including toll-like receptor (TLR) agonists (115, 116), lymphotoxin β receptor (LTβR) agonists (117, 118), and stimulator of interferon genes (STING) agonists (119, 120).

Therapeutic vaccination shows significant potential for inducing TLS, as evidenced by clinical observations and preclinical models. In patients with high-grade squamous intraepithelial lesions (HSILs), administration of human papillomavirus (HPV) vaccines elicited mature TLS formation within regressing lesions (121), suggesting vaccine-mediated remodeling of the tumor microenvironment facilitates lymphoid neogenesis. Similarly, combinatorial therapy using pancreatic tumor vaccines (GVAX) with cyclophosphamide induced TLS development in the majority of treated pancreatic cancer patients (122). Recent advances include a nano-vaccine platform incorporating Epstein-Barr virus nuclear antigen 1 (EBNA1) with a dual adjuvant system of Mn²^+^ and cytosine-phosphate-guanine (CpG) formulated in tannic acid, which achieved significant suppression of nasopharyngeal carcinoma progression in murine models through TLS potentiation (123). These collective findings position tumor vaccines as a promising therapeutic strategy for deliberate TLS induction.

#### 5.2.2 Suppression of “Immunosuppressive TLS”

In RCC, TLS have been observed to correlate with unfavorable patient prognosis in some instances. Similarly, TLS have been associated with poorer clinical outcomes in various chronic infectious and autoimmune diseases. These findings suggest the potential therapeutic value of suppressing TLS that contribute to adverse prognosis. However, the majority of current research efforts have focused on promoting TLS formation and maturation, with relatively few studies investigating strategies for TLS suppression. Particularly in the context of cancer, the precise mechanisms underlying TLS formation remain incompletely elucidated. Drawing upon established approaches for TLS induction, potential suppression strategies may include targeted inhibition of LTi-like or LTo cell proliferation and activation, disruption of interactions between LTi-like and LTo cells, suppression of cytokine and chemokine secretion, or direct intervention against chemokines such as CCL19, CCL21, and CXCL13 through administration of specific antibodies to inhibit lymphocyte aggregation. Published studies have demonstrated that direct depletion of T follicular helper cells or B cells can effectively suppress TLS formation (124), suggesting that selective depletion of specific tumor-infiltrating lymphocyte subsets, particularly exhausted CD8^+^ T cells, within renal cancer-associated TLS may help alleviate immunosuppressive conditions. HEVs play a critical role in TLS development, suggesting that blocking HEV formation through anti-angiogenic agents or suppressing PNAd expression may represent an alternative approach for TLS inhibition. Furthermore, genomic analyses of TLS-positive ccRCC have identified frequent mutations in genes including *VHL*, *PBRM1*, *SETD2*, and *MTOR* (44), which may serve as potential biomarkers for TLS-targeted therapeutic strategies.

Recent studies have identified a novel regulatory mechanism of TLS involving lysophosphatidylserine (LysoPS) produced by activated B cells, which suppresses TLS formation by disrupting B cell-B cell or B cell-T cell interactions through the Gα13 signaling pathway of G protein-coupled receptors (125). In studies of primary biliary cholangitis (PBC), administration of the sphingosine-1-phosphate receptor (S1PRs) modulator FTY720 in mouse models was found to inhibit TLS and ameliorate cholangitis (126). These findings demonstrate that in-depth investigation of TLS formation mechanisms and functional characteristics across different disease contexts could provide critical insights for developing targeted TLS therapeutic strategies to achieve more effective treatment outcomes. Furthermore, studies have reported that *Hhep* promotes TLS formation and function (37), suggesting the potential existence of microbiota capable of inhibiting TLS development that may be discovered in future research.

## 6 Discussion

### 6.1 Existing challenges

The substantial potential demonstrated by TLS as both prognostic biomarkers and therapeutic targets is poised to play a pivotal role in future cancer treatment paradigms. However, the clinical translation of TLS-based applications continues to face numerous challenges.

First, in RCC, TLS as prognostic biomarkers still exhibit certain contradictions. Heterogeneous TLS features in renal cell carcinoma may predict divergent prognostic outcomes; however, in other cancers such as lung cancer (64, 65), ovarian cancer (66), colorectal cancer (42, 57), and bladder cancer (67, 68), TLS appear to be associated with improved prognosis and enhanced response to immunotherapy. It is noteworthy that while the findings from Masuda et al. suggested that the presence of tumor-distal TLS is associated with poor prognosis, the TLS they observed were exclusively early-stage TLS, and no secondary follicle-like TLS (SFL-TLS) with germinal centers were detected (44). On one hand, this relates to the heterogeneity TLS demonstrate in terms of composition, maturation status, spatial distribution, and density. On the other hand, although specific markers of TLS have been extensively studied, a unified classification and evaluation criterion for TLS remains lacking. For instance, different studies employ varying diagnostic criteria to determine the presence of TLS, and the characteristics of included TLS are difficult to standardize. Even the current definition of “mature TLS” lacks a set of uniform parameters, resulting in insufficient comparability of data across different studies.

Secondly, existing research collectively suggests that the composition, maturity, spatial distribution, and density of TLS in renal cell carcinoma may be associated with patient prognosis and response to immunotherapy, highlighting the potential of TLS as both a prognostic biomarker and a therapeutic target. However, current findings on TLS in RCC primarily stem from single-center, retrospective studies, which are susceptible to influences from regional epidemiological characteristics, surgical approaches, and baseline differences in the immune microenvironment, leading to selection bias and recall bias. There is a scarcity of multicenter, large-sample cohort studies. Furthermore, variations exist across studies in aspects such as the source of TLS tissue samples and detection protocols, patient inclusion and exclusion criteria, baseline characteristics, endpoint definitions, control of confounding variables, and follow-up duration. These factors collectively contribute to discrepancies in conclusions across different cohorts, limiting the comparability of results and the strength of causal inference. It is particularly noteworthy that many studies still focus on intermediate endpoints such as immune infiltration and TLS structural characteristics. Evidence centered on clinical endpoints like rehospitalization rates or functional recovery outcomes, with adequate control for confounding, remains relatively limited. Concurrently, whether TLS-related indicators can provide stable, reproducible, and interpretable clinical benefits requires validation within prospective study designs. Furthermore, TLS-targeted therapies present both therapeutic potential and concomitant risks. Although therapeutic induction of TLS is regarded as a novel approach to enhance anti-tumor immunity, its clinical translation faces multiple challenges: ① The induction of TLS formation and maturation may potentiate autoreactive T-cell and B-cell responses in other tissue compartments. Studies have demonstrated that TLS formation following PD-1 blockade may correlate with autoimmune diseases, suggesting TLS induction could trigger immune-related adverse events (127). ② Certain strategies, such as the administration of stromal cells, cytokines, or chemokines, may systemically activate the immune system rather than specifically targeting TLS within the tumor microenvironment, potentially leading to off-target effects. ③ Existing murine TLS models exhibit notable differences from clinical TLS models. While TLS can be rapidly induced in murine models, the development of human tumor-associated TLS predominantly relies on prolonged, chronic inflammatory conditions. Moreover, the reproducibility of successfully induced murine TLS in human tumors remains uncertain.

### 6.2 Future research directions

Based on the aforementioned numerous challenges, we should first establish a scientifically rigorous and reproducible standardized definition and evaluation system for TLS in the future. This system should clearly define TLS and provide detailed classification methods, establishing a multidimensional evaluation standard encompassing TLS composition, maturity, spatial distribution, and density. This will ensure conceptual consistency across studies, construct a standardized TLS definition and evaluation framework, unify research criteria, enhance the comparability of results from different studies, and thereby clarify the research direction for related mechanisms, providing a theoretical foundation for further investigation into TLS-related mechanisms. In the future, we can fully integrate technologies such as spatial transcriptomics, single-cell sequencing, multiplex immunofluorescence, clinical models, and radiomics models in combination to further dissect the differences among different types of TLS at the levels of cellular composition, functional states, and signaling pathways. Particular focus should be placed on kidney cancer-specific pathways such as VHL-HIF, PI3K–mTOR, and NF-κB, to reveal the mechanisms by which genetic or epigenetic factors precisely regulate the TLS formation process in renal cell carcinoma. Concurrently, immunocompetent orthotopic xenograft tumor models or patient-derived tumor organoid/patient-derived xenograft models can be utilized to explore the specific mechanisms by which TLS influences the prognosis of renal cell carcinoma patients (128, 129). Secondly, conducting multicenter, large-sample prospective clinical studies to validate the relationship between TLS heterogeneity and patient prognosis in renal cell carcinoma, and systematically evaluating the associations between TLS composition, maturity, spatial distribution, density, and patient survival outcomes as well as responses to immunotherapy. Such research will help clarify whether TLS can serve as an independent prognostic biomarker or therapeutic target for renal cell carcinoma. In the future, TLS can be incorporated into real-world studies. For instance, in clinical trials related to immune checkpoint inhibitors (ICIs), characteristics of TLS such as composition, maturity, spatial distribution, and density can be included as predefined or exploratory biomarkers to dynamically assess the formation and remodeling of TLS before and after treatment, as well as their relationship with therapeutic efficacy and immune-related adverse events, thereby gaining a better understanding of the functional role of TLS in treatment response. TLS-targeted therapeutic strategies should be further optimized in the future, such as developing targeted delivery systems to precisely regulate chemokine gradients in the tumor microenvironment and avoid off-target effects associated with systemic drug administration. Promising biomaterials currently under investigation, including nanoparticles, biological scaffolds, and hydrogels, have demonstrated considerable potential. Furthermore, exploring combination regimens of TLS-targeted inducers with ICI therapy may contribute to the development of next-generation immunotherapeutic approaches, thereby improving ICI response rates and patient survival outcomes. Notably, at the level of clinical translation, future research must cautiously evaluate therapeutic strategies aimed at inducing or remodeling TLS, clearly delineating which TLS possess genuine antitumor functions and which may promote immunosuppression or drug resistance, thereby providing a foundation for precision immunotherapy.
